# A Matter of Trust: Confidentiality in Therapeutic Relationships during Psychological and Medical Treatment in Children and Adolescents with Mental Disorders

**DOI:** 10.3390/jcm13061752

**Published:** 2024-03-18

**Authors:** Johanna Xenia Kafka, Oswald David Kothgassner, Anna Felnhofer

**Affiliations:** 1 Department of Pediatrics and Adolescent Medicine, Division of Pediatric Pulmonology, Allergology and Endocrinology Medical University of Vienna, 1090 Vienna, Austria; johanna.xenia.kafka@univie.ac.at; 2 Department of Child and Adolescent Psychiatry, Medical University of Vienna, 1090 Vienna, Austria

**Keywords:** confidentiality, autonomy, therapeutic relationship, child and adolescent psychology

## Abstract

**Background:** Confidentiality is a crucial ethical principle in therapy, particularly for children and adolescents, yet their perception of it remains understudied. We aimed to explore minors’ perspectives and attributions on confidentiality in psychological and medical treatment. **Methods:** We interviewed 11 pediatric patients aged 7 to 15 and used reflexive thematic analysis to analyze their responses. **Results:** Four main themes were extracted from the data: (1) confidentiality and uncertainty regarding what information will be shared with clinicians and parents; (2) consequences of breaching confidentiality, encompassing breaches of confidentiality in the past and their negative effects on interactions with parents and health professionals; (3) exceptions to confidentiality, including understanding the limits of confidentiality; and (4) autonomy and self-determination, reflecting the desire for involvement in medical decisions. **Conclusions:** Explanations about confidentiality rules and limits, especially with younger children, are crucial. This is particularly important because it is fundamental to promote children’s development and self-determination through increasing autonomy, as well as to provide a sense of security and respect through transparent rules. A single educational session on confidentiality at the outset of therapy is insufficient; ongoing conversations are needed to reinforce understanding and promote autonomy.

## 1. Introduction

The concept of confidentiality can be traced back as far as to the Hippocratic Oath which states that “what I may see or hear in the course of treatment […] I will keep to myself, holding such things shameful to be spoken about.” (see [1]). As such, confidentiality is rooted in the ethical principles respect for privacy and respect for autonomy [2,3] and is considered a fundamental prerequisite for an effective doctor–patient or psychologist–patient relationship [4]. Confidentiality has been defined as the expectation that information disclosed in a sensitive context will not be revealed to a third party without prior consent [5]. In contrast, privacy describes the right of an individual to be protected against intrusion into their personal space [5]. The 4-dimensional model of privacy describes confidentiality as informational privacy and contrasts it with psychological, social, and physiological privacy [6]. Within this model, psychological, social, and physiological privacy pertain to the individual’s ability to control one’s social interactions, bodily space, and judgement by others (see [7]). As a consequence, the concept of confidentiality illustrates a professionals’ respect for the privacy of their patients.

### 1.1. Breaching Confidentiality in Minors

The maintenance of confidentiality and its limits has been posing ethical dilemmas to psychologists, psychotherapists, and related health professionals for decades (see [8,9,10]). Those providing medical care to children and adolescents are faced with challenges that differ from those encountered in adult care [11,12]. In contrast to adults, children and adolescents are usually not yet legally autonomous [13], and they tend to be more emotionally and physically vulnerable than adults [14]. Therefore, the support of autonomy and protection in pediatric mental health treatments has to be balanced [15]. Furthermore, the past studies [16] suggested that young patients may be more concerned about confidentiality than grown-ups. Accordingly, medical care providers have repeatedly expressed uncertainty regarding when to breach confidentiality. This uncertainty becomes especially pronounced in the context of adolescent risk behaviors such as drug use, sexual behaviors, and suicidal ideation (e.g., [8,11,12]). In light of this uncertainty, the investigation of children’s and adolescents’ understanding of confidentiality is vital. To be effective, medical care must explore and integrate minors’ perspectives and past experiences with confidentiality and potential breaches of it.

### 1.2. Sequela of Confidentiality Issues in Minors

The research has consistently been demonstrating that concerns about confidentiality entail significant consequences for health behaviors in adolescent patients [17,18]. Minors articulate fear regarding their physician informing their parents about the reason of the consultation, particularly if sensitive issues such as contraception, sexual activities, or drug use are involved (e.g., [17]). This leads to an increased reluctance to seek medical care and may result in forgoing treatment altogether [16,17,19,20]. As a consequence, youth are more likely to engage in risky behaviors [17] and are at higher risk of developing mental disorders in the longer term [21]. Conversely, education about confidentiality increases adherence, trust, and acceptance of medical treatment in young patients [22].

These findings underline the importance of informing underage patients about confidentiality. Even brief conversations appear to increase adolescents’ willingness to disclose sensitive topics to mental health care providers [23]. Although young patients seem to have some understanding of the limits of confidentiality, they tend to lack clarity on the exact topics that physicians may disclose to parents [23,24]. Even more concerning, Zucker and colleagues [18] found that most adolescents have never had a conversation about confidentiality with their mental health provider at all.

### 1.3. Parents and Confidentiality

Compared to medical care in general, the mental health sector faces specific challenges concerning confidentiality. Since interventions in this field emphasize family orientation, parents are usually more involved in the treatment [3]. Furthermore, there is an increased risk of stigma, and as a result, information may be perceived as more sensitive and may be shared differently by those affected. Ultimately, the effectiveness of psychological therapy hinges on confidentiality. Correspondingly, a recent systematic review [25] demonstrated that adolescents perceive trust and confidentiality in therapeutic relationships as a significant facilitator—or barrier in the case of a lack thereof—to consulting mental health care services (see [26,27]).

To date, most studies investigating confidentiality in underage populations focus on adolescents. Arguably, younger patients’ needs differ from those of adolescents. Given that young children typically rely more heavily on their parents, the research on this demographic’s relationship with confidentiality is warranted. The few existing findings suggest that, even at a young age, confidential communication with medical staff is crucial for children [28]. Although they seem to have some understanding of the concept at elementary school age [29], children struggle with outlining when and which information is shared with parents [24].

### 1.4. Study Objective

The prior research has demonstrated that minors deem confidentiality crucial (e.g., [16]). Furthermore, being informed about the scope of confidentiality in therapy improves medical treatment adherence and acceptance (e.g., [22]), while confidentiality concerns hinder the therapeutic process (e.g., [16,17,19,20]). However, it remains unclear how children and adolescents perceive confidentiality in mental health care. Expanding the research to include younger children is essential to understanding their attitudes and perceptions In view of the limited data, we opted for qualitative semi-structured interviews to understand young patients’ perspectives on confidentiality in therapy, with the ultimate goal of addressing their needs, respecting their rights, and ensuring a child-friendly approach to medical and psychological care.

## 2. Materials and Methods

### 2.1. Participants and Procedure

We recruited our participants from June 2016 to February 2017 at a Day Clinic for Paediatric Psychosomatic Medicine at the Vienna General Hospital, Medical University of Vienna. The patients were approached after a pre-consultation with the clinical staff. Children and adolescents were eligible if their language skills, intellectual capability, and emotional stability allowed for a face-to-face interview with a relative stranger. All the pertinent details were deliberated beforehand with the treating team, and a joint determination was reached regarding the clinical manifestation of the inclusion criteria. The potential participants were approached by the researchers. Other inclusion criteria were ongoing inpatient treatment at the Day Clinic as well as an age between 6 and 15 years. An effort was made to talk to the child/adolescent and the caregiver in person to tell them about the study and ask for their willingness to participate. Most of the approached families (82%) gave assent and consent. Some declined simply by stating that they had no interest, and a few stating concerns about data protection.

Ethical approval was granted by the local institutional review board (vote number 1044/2016). Written consent had to be given prior to participation both by the parents and by the child or adolescent. No remuneration was advertised or offered. Upon providing informed consent, the caregivers received a short sociodemographic questionnaire that they could either fill out immediately or take home and return later. Furthermore, we conducted semi-structured face-to-face interviews to enable us to talk to minors about their views on confidentiality in the therapeutic relationship in the context of psychological and medical treatment. Each participant was interviewed individually, face-to-face, by the first author, who introduced herself as a psychologist who was new to the clinic and was interested in their valuable opinion on confidentiality and related topics. The interviews took place in whichever room of the Day Clinic was available and allowed for an undisturbed and relatively comfortable interview situation. Before switching on the recording device, the participants were informed that everything said during the interview would be treated confidentially unless information was revealed that gave rise to the assumption that the participant or any other person was in danger of serious harm. If a participant discussed something potentially threatening (e.g., suicidal thoughts), the interviewer asked for more details in order to decide if it was necessary to contact clinical staff. This incident never occurred in the current study. If participants disclosed personal secrets during the interviews, they were only transcribed if the participants explicitly agreed to this.

After starting the audio recording, the participants were provided with a pile of cards with various topics written on each card. The topics were chosen based on the prior literature [23] and with the intention to present the participants with a range of subjects that they may potentially intend to keep a secret from others, especially caregivers (e.g., parents). In total, 37 items were presented ranging from smoking, taking drugs, and shoplifting to suicidal thoughts and other self-harming or aggressive behavior. The participants were asked about their opinion on whether this would be disclosed to the parents by the psychologist/therapist/doctor after a consultation regardless of the children’s wishes. While the interviewees were sorting the cards into piles, they were asked to share their thought processes. Upon completion, the participants were asked to talk about what confidentiality meant to them, what kind of experiences they had with regard to confidentiality, if there were any exceptions, and if there was something they wished to change. It is important to note that the interviewees were asked to share their opinions and feelings freely and that they were reminded criticism would not be shared with the Day Clinic staff. The participants were encouraged to not only share experiences they made during their current inpatient treatment, but to also refer to previous experiences. At the end, the participants were given the chance to add something that seemed important to them but had not been asked or talked about during the interview.

After completing the interview and as a sign of appreciation for their participation, the children and adolescents could choose a game to play with the interviewer if they wished. The interviews lasted for approximately 20–30 min. One interview was divided into three parts because the child was struggling to keep up attention. The first interviewee was interviewed twice with the intention to see if anything had changed a few days later. It soon became clear that this could easily become overwhelming for some of the children, especially the younger ones. Therefore, we chose to conduct just one interview session per participant.

All potentially identifying details such as names of patients or staff members have been anonymized in the process of transcribing the interviews. This was implemented not only to protect the anonymity of the interviewees and named health professionals but also to prevent conflicts of interest whilst analyzing the data.

### 2.2. Analysis

For the analysis of the audio recorded interviews, we used reflexive thematic analysis as outlined by Braun and Clarke [30,31]. As a first step, the interviews were transcribed verbatim by the first author. This process was also used to become familiarized with the data. After that followed an intensive process of coding the data by the first author. The theme development and review were performed by the first author in consultation with the co-authors. We used an inductive approach and based our analysis on the content of the transcripts and, therefore, had no pre-existing framework or coding scheme. First and foremost, we intended to give voice to minors and their feelings and conceptions of confidentiality. However, of course, we have to acknowledge that our analysis is influenced and shaped by our own experiences as mental health professionals and researchers in the field of clinical psychology. Our intention to give voice to minors in this specific topic also reflects our presumptions that this is important for minors and that this subject constitutes a partly overlooked topic in the research as well as in everyday practice. Given that a power dynamic existed between the researcher and the patient, coupled with the fact that the researchers belong to the professional groups under investigation, this undoubtedly exerts a distorting influence on the willingness of the interviewed patients to provide information. Moreover, the interviewers, being part of the relevant professional disciplines, are acutely aware that in the medical–therapeutic process, breaches of confidentiality may occur, either unconsciously or necessarily. This awareness has undoubtedly informed the design and analysis of the study.

## 3. Results

### 3.1. Participant Demographics

Of the 11 participants included in this study, 6 identified as girls and 5 as boys. The participants’ ages ranged from 7 to 15 years. More participant demographics are displayed in Table 1. The parents stated various reasons for the admission of their child, ranging from somatic illnesses to psychiatric disorders.

### 3.2. Interviews

**Figure 1:**
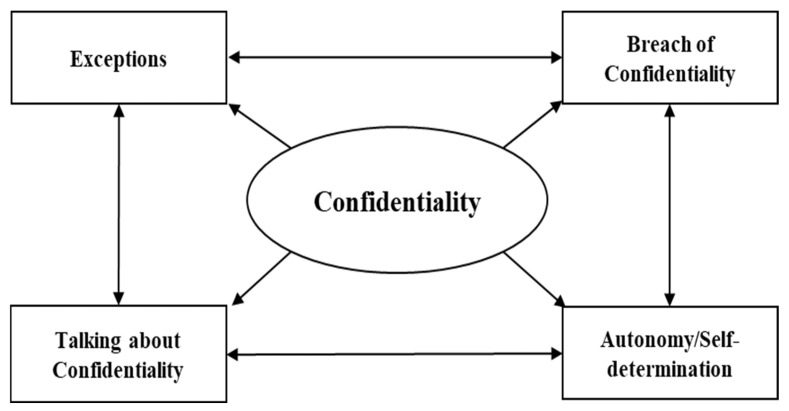
Four thematic topics resulted from interviews and their interactions.

Based on the inductive thematic analysis of the transcribed interviews, we organized our data into four themes: (1) talking about confidentiality, (2) consequences of breaching confidentiality, (3) the exception that proves the rule, and (4) autonomy/self-determination. These four categories are connected, and all are equally important. Figure 1 visualizes their connectedness. An educational talk about confidentiality cannot miss the exceptions and the consequences of breaching it. Being serious about confidentiality is also an important part of (growing into) autonomy and self-determination.

#### 3.2.1. Talking about Confidentiality

At the beginning of each therapeutic relationship with a mental health professional, the children and adolescents are usually informed about confidentiality and its boundaries. The participants in our study reported experiencing this educational talk quite differently. On the one hand, being educated about the specifics surrounding the issue confidentiality can be experienced as a means of building trust.

Participant: “For example, some therapists have told me what I am telling them, they are not allowed to pass it on.”

Interviewer: “And how is it for you when they tell you that?”

Participant: “Well, I can trust them.” (Interview 8)

On the other hand, talking about confidentiality can also be experienced as something that has to be carried out briefly at the beginning of a therapeutic relationship and is quickly forgotten afterwards.

Participant: “(sighs) (...) I think it will be touched on very briefly at the beginning, but not really.” (Interview 1)

There is also discontent about the fact that these educational talks do not include detailed information about what and in which instances information is exchanged within the team of clinical staff members or with external (mental) health professionals.

Participant: “What is the team?

Interviewer: “The doctors, the other psychologists, the social pedagogues

Participant: “Do I actually know them?

Interviewer: “Who?

Participant: “The team

Interviewer: “You know most of the team. You know [name of therapist].

Participant: “I actually find it very unfair if information about me is passed on to people I have never seen or known. I don’t think that’s okay even if I know the person. I don’t like that something is being said behind my back. Especially if something is really important for me somehow like the thing with the medication. Everyone probably already knows that by now, because [name of therapist] chatted. but yes. (Interview 10)

The same holds true for conversations between therapists and parents. Minors are curious about what is talked about, but at the same time, they know with almost certainty that they have no say in what is talked about and what is not.

Participant: “I’ve never asked, and I don’t think they’ll tell me what they talked to my mother without me.”

Interviewer: “But would you like to know?“

Participant: “Yes.” (Interview 4)

#### 3.2.2. Breaches of Confidentiality and Their Consequences

Some participants stated that they trust their therapists to keep their secrets. Others, however, shared their experiences regarding therapists breaching confidentiality. Mostly, they noticed the breach because parents told or asked them about information the participant had only revealed to the therapist and that was not intended to be shared with the caregivers.

Participant: “Well, they [the parents] just tell me what they learned. And at the same time I notice that a lot is being told, a lot is being told.” (Interview 1)

Such an experience can lead to a wide range of emotions and reactions. One consequence being the refusal to disclose potentially important information to a therapist.

Participant: “It’s actually very important to me, because I think that’s the discussion with the psychologist and not with my parents, maybe I’ll tell the psychologist things that my parents don’t know and sometimes it just happens I’ll come home from [Name of therapist] when they had an appointment and my mother says, [name of adolescent], what did you actually tell [name of therapist] and all of that is just completely uncomfortable for me and I don’t want that. I just want people to be discreet about that.” (Interview 10)

Another strategy is one we named “strategic story telling”. This is an example for the way minors turn something they feel negative about into something useful for themselves. Sometimes, minors start thinking about what to share and what to better keep for themselves. Therapists unknowingly become messengers to pass on information that one finds hard to share personally but wants others to be informed about.

Participant: “Yes, there are things that you can’t really talk to your parents about, for example, then I talk to [name of therapist] about them and then the parents know too.” (Interview 1)

Participant: “Yes. Oh, that is already practical. It’s just really good the way I currently have it, when one has two, just [name of therapist], where you know it is passed on, then I don’t have to talk to the parents about it and (...) and my external [therapist] where I know I can say what I want it will never be told.” (Interview 1)

Furthermore, a sort of resignation often emerges that a breach of confidentiality is something that has to be expected as a minor and not much can be done about it. This leads to minors keeping relevant information secret to protect themselves from being exposed.

Participant: “I don’t have a big problem with it now as I said like I said before then I don’t tell them [therapists] so much.” (Interview 11)

However, (mental) health professionals are not the only the ones breaching confidentiality. Parents may also share information about their child that is considered to be confidential or embarrassing by the child.

Interviewer: “OK. Do you mean your parents tell the psychologist or doctor something or vice versa, who is telling whom what you don’t want?”

Participant: “The parents, my mom, my dad could tell something I don’t like that they tell.” (Interview 3)

#### 3.2.3. The Exception That Proves the Rule

Our interviewees valued confidentiality in general as a very important component of the therapeutic relationship and the ability to keep a secret as something that is very important in everyday life as well. However, confidentiality always comes with certain exceptions. In almost all interviews, minors expressed an understanding for exceptions. The possible reasons were mostly along the lines of doing something extreme or something that constituted a serious threat to one’s own life or health. Examples were taking drugs, having suicidal thoughts, or doing something illegal.

Participant: “Can be when, for example, I am not me.”

Interviewer: “What do you mean if you are not you?”

Participant: “If I take drugs, for example, it will be passed on immediately, but do I? No.” (Interview 11)

Or as another participant expressed it:

Participant: “[pauses] Yes, they are either completely illegal, or they cause problems for others. otherwise I think things shouldn’t be passed on. except now all these extreme things.” (Interview 1)

Participant: “Yes, if there is really an acute danger for something, what do I know suicide or something. but otherwise no.” (Interview 1)

There was also a certain understanding for the fact that sometimes it is necessary to breach confidentiality to be able to obtain the help that one needs, like telling a doctor about medical symptoms or hearing voices so that medication can be prescribed, and possible harm can be prevented.

Participant: “Hm [pauses] so that you get help.”(Interview 8)

Similarly, there was also some appreciation for the fact that the mental health professional may disclose some information in order to mediate between parents and children.

Participant: “And not wanting to live at home. With this I would say that you should talk to the parents and so they can understand.” (Interview 9)

Surprisingly, there were themes mentioned as exceptions by the children and adolescents that seemed morally wrong to them (e.g., lying to parents). This is particularly interesting because other interviewees mentioned the same examples as things to keep confidential no matter what.

#### 3.2.4. Autonomy/Self-Determination

Our interviewees did not consider age as a relevant factor with regards to confidentiality. It was argued that lived experiences speed up the process of maturation in certain fields of life related in some way or another with the disease or disorder.

Participant: “Yes that it is just that you [pauses] That you grow up mentally faster through such things as I said.” (Interview 1)

It was also expressed that one must continuously defend their rights against parents and/or mental health professionals. The importance of voicing one’s own wishes was stressed by our participants, as well as the impossibility to realize one’s wishes if one is not able to talk for themselves (e.g., when a medication is applied).

Participant: “I just think, if the child is smart enough and really has his own will, if it really says explicitly, he doesn’t want, that it keeps getting ignored, because that’s a human right, that’s true, that’s true, it’s really in the law. Then it will probably not be passed on unless it is dangerous for the child. Or and if the parents just don’t want that and are just stubborn, as in my case, then maybe the 15-year-old will have to take that too, just because he doesn’t stand up enough for his or her will.” (Interview 10)

The wish for autonomy was also voiced as a form of “my body, my choice” when it came to whether or not to take prescribed medication, for example. The perceived lack of control over decisions concerning one’s health and life is experienced as particularly frustrating.

Participant: “(...) because the parents must know how the child is doing. and everyone obviously always believes that the child is the parents. that the child has no opinion of their own, but that the child should always be of the opinion of the parents.” (Interview 10)

### 3.3. Card Sorting

Sorting the pile of cards was a somewhat difficult task for some of our participants. Some explained that it is not easy to imagine things that they had not yet experienced themselves. Some found it difficult to imagine a hypothetical child or adolescent with a certain problem. Younger participants struggled more; particularly the interviewees with ASD expressed difficulties. Of note was that participants explained that there was a difference between how they sorted the pile according to whether they believed that the therapist would disclose/not disclose the information and how the pile should have been sorted according to their values and beliefs.

There was only one item on which all participants unanimously agreed: telling a therapist that the individual had been hit by another kid would certainly be disclosed. The second most often agreed upon item was “not wanting to take medication”. Here, eight participants stated the therapist would tell parents about this circumstance, while two participants were not sure about the outcome. Among the items cited most often in the category of non-disclosure by the therapist were (with 7 nominations each) talking badly about their parents, disclosing a secret of their parents, and revealing a secret of one’s own. The complete number of nominations is presented in Table 2.

The topics that the participants were most often not sure about (with 5 nominations each) were shoplifting, nude pictures, and having hurt others. This indicates that even though it is mandatory that minors must be informed about confidentiality and its limits, there is still confusion about the practical implications.

### 3.4. Summary of Results

Summarizing these findings, not all minors report receiving satisfactory information about confidentiality and its boundaries at the onset of therapy. Additionally, there is often uncertainty regarding the disclosure of private information to other professionals or parents. While minors typically trust therapists to maintain the confidentiality of their patients’ secrets, breaches of confidentiality (e.g., with parents) are frequently observed. This uncertainty and breach of trust may prompt minors to reconsider what information they share with therapists or experience feelings of resignation. Nevertheless, minors acknowledge situations where breaching confidentiality may be necessary in the therapeutic context, particularly in cases of potential harm to the patient. Nonetheless, it remains crucial for minors that their wishes and needs, especially concerning medication, are carefully considered. In the card sorting task, it was evident that certain sensitive topics (e.g., sexuality and criminal activities) regarding the boundaries of confidentiality need to be communicated more clearly. However, when the child is a victim of physical altercations, there is a greater expectation that this information will be disclosed.

## 4. Discussion

We interviewed a diverse group of patients currently receiving treatment at a Pediatric Psychosomatic Medicine Unit for various reasons. To our knowledge, this is the first study to focus on the perspectives and attitudes of children and adolescents with mental health issues in a healthcare setting. Based on the participants’ verbal responses and thorough inductive thematic analysis, four main topics emerged, which are discussed further below: (1) conversations about confidentiality, (2) consequences of breaching confidentiality, (3) the exception that proves the rule, and (4) autonomy/self-determination.

### 4.1. Understanding Confidentiality

Overall, discussions about confidentiality with mental health care professionals prior to the start of treatment were perceived as a means of building trust by most participants. Therefore, it is troubling that some interviewees mentioned not remembering such conversations ever occurring. While this aligns with previous findings [18], the underlying causes remain unclear. It is possible that those affected were never informed about confidentiality. The studies on children’s consent capacity indicate that younger children are more likely to be presumed incompetent by medical professionals, potentially excluding them from shared decision-making processes [32]. Additionally, confidentiality ranges among the most complex and difficult legal and ethical issues, which are additionally complicated by differing institutional practices, personal beliefs, and professional codes [8]. As a result, medical care providers have repeatedly been found to be unsure about the particularities of confidentiality [9]. This may contribute to the difficulty of conveying key points to young patients, particularly as young children may struggle to grasp the concept if it is explained only abstractly [24]. When information is presented explicitly and relates more closely to a child’s daily life, it is easier for them to understand and remember.

Generally, our participants demonstrated some understanding of the limits of confidentiality (as assessed by the card sorting task). However, consistent with the prior research [23,24], there was considerable uncertainty about which topics would be disclosed to others and which would not. In addition to that, children were unsure about which information was shared with the parents and with the medical team. The relevant literature lacks an exploration of confidentiality within larger mental healthcare teams, involving professionals from diverse backgrounds, i.e., nurses, psychologists, and doctors [3]. On the one hand, different team members may have varying perspectives on confidentiality, resulting in differing actions toward minors [33]. Ultimately, children and adolescents are curious about what information is shared and desire more detailed information about it.

### 4.2. Need for Autonomy

Increasing autonomy is particularly important to promote the development and self-esteem of minors [34]. Of major concern, is the finding that participants were almost certain that they would have no say in what was said about them. This was markedly contrasted by the strongly voiced need for autonomy. Instead of age, children and adolescents in this study underlined aspects of cognitive and emotional maturity as an essential evaluation criterion for whether they should be deemed competent to decide for themselves. The past research shows that the older minors are, the stronger the need for autonomy with regards to mental health issues; for example, in a sample of 14–24-year-olds, particularly those above 18 years believed that all care should be confidential [18]. This is reflected in the approach of Fisher and colleagues [35], who prioritize a weighing process in their goodness-of-fit ethics model, focusing on balancing autonomy and risks, aligning with the principle of the mature minors.

Balancing the need for autonomy against the need to protect is particularly difficult in pediatric care as “[the child’s] very age and vulnerability, as a non-adult, mean that their entitlement to unfettered respect for their right to autonomy, self-determination and independence is unlikely to be fully implemented” ([14], p. 264). Following the protection–autonomy model [36], mental health care providers have to balance autonomy and the protection of the child in pediatric treatment. This model also emphasizes the decisional capacity that allows professionals to determine the degree of support for the child’s autonomy and the level of protection required. These two poles are not mutually exclusive but rather are derived from the developmental, cognitive, emotional, and medical needs of the patient [15]. It is all the more important that mental health care providers make an effort to routinely, repeatedly, and developmentally appropriately explain the protections and limits of confidentiality to reach the best possible grounds for a trusting therapeutic relationship [23,35].

In addition, children’s autonomy in health-related decisions is also influenced by family and cultural factors. Healthcare professionals have a dual role to play: they must support the child’s autonomy and, at the same time, promote parental health-related decisions in the best interest of the child, which can be challenging when parental decisions are not unanimous (e.g., separation of parents, conflicts between parents). Another complex situation may arise from cultural factors, as parents with different cultural norms may have different views on the autonomy of their own children [15]. This can lead to the protective factor no longer being in balance with the necessary autonomy of the child.

### 4.3. Breaches of Confidentiality

Along with positively lived therapeutic relationships, past experiences with breaches of confidentiality were also reported by our participants. In most cases, those affected learned about the violations by chance, for instance, when parents mentioned information they had heard from the psychologist or psychotherapist. Remarkably, our participants expressed a sophisticated understanding for exceptions. Among other circumstances, they identified being a threat to oneself or others as a situation in which mental health care professionals would be obliged to breach confidentiality. However, they desired clear communication about potential breaches beforehand. Otherwise, the breach may harm the therapeutic relationship and the minor’s health.

The prior findings have already demonstrated the detrimental effects of a shaken confidence, ranging from a reluctance to disclose potentially relevant health information to completely forgoing medical treatment [16,17,18,19,20]. Our study adds additional support to these findings and extends them to the particularities of a therapeutic relationship with psychologists and psychotherapists. Within this context, the refusal to disclose potentially important information or strategic storytelling may lastingly impair the opportunity for a therapeutic rapport characterized by trust and respect. The resignation observed among some of our participants is particularly worrisome with regards to treatment outcome. The data suggest that keeping secrets to oneself is associated with increased burden such as stress, worries, and decreased well-being and adjustment [37]. Hence, apart from the necessity of building a therapeutic treatment on honesty, openness and trust, confiding in another person may in itself be an alleviation of mental and emotional strains.

Another interesting aspect that arose from the interviews was that not only were children and adolescent disappointed by the therapist unsolicitedly sharing information but they also mentioned that they have had negative experiences with their parents as well. Hence, parents may, in their children’s view, also violate their privacy. Particularly, this may concern the minor’s psychological privacy and social privacy that, according to Burgoon’s [6] 4-dimensional model of privacy, pertain to the protection of attitudes from unwanted disclosure and control of one’s social interactions. As demonstrated by the prior research [38], secrecy towards parents may have a specific developmental function: Particularly in the period of adolescence, increased secrecy may serve the development of emotional autonomy, i.e., individuation and expansion of the autonomous self. This should be taken into account in the process of therapy, and the degree to which information is disclosed by the psychologist or therapist should always be weighed against the type of relationship between the child and their parents [18]. Furthermore, it was observed in the card sorting task that clear communication regarding the boundaries of confidentiality is also crucial for socially sensitive (e.g., sexuality) and legal (e.g., criminal activities) topics, as these may be relevant to some children in their lived experiences. While age may play a role, it is expected that these topics will increasingly become evident among minors as well.

### 4.4. Practical Implications

Our results demonstrate that minors desire discussions about confidentiality boundaries and that they express uncertainty regarding the protection by confidentiality rules. They can feel betrayed, hurt, or angered if information is disclosed without their consent. At the same time, young patients acknowledge that not all information can remain confidential. They recognize a potential need to share details with parents or other (mental) health professionals. In most cases, an initial educational talk between a minor and a mental health professional may not suffice. Based on our findings, it is recommended to frequently discuss the boundaries of confidentiality while also emphasizing what information will be protected by it. Both the goodness-of-fit ethical model [35] and the protection–autonomy model [36] indicate that boundaries of confidentiality exist prior to therapeutic interventions. These boundaries should regularly be readjusted according to the child’s cognitive capacities and emotional needs. Mental health care providers should have procedures in place to address parental inquiries regarding information about their children. Necessary breaches of confidentiality should be carefully considered, prepared for, and explained (e.g., [15]).

## 5. Limitations

This study is not without limitations. A larger sample size would have likely improved the quality of our results, facilitating a more comprehensive analysis of developmental psychological influences across different age groups. With a larger sample size, we could have examined the main themes in greater detail and examined potential differences in their importance across age groups. Additionally, comparing clinical presentations (e.g., internalizing vs. externalizing disorders) and exploring specific thematic nuances would have been feasible.

The descriptive results of the opening task, sorting cards into piles of what will be kept confidential, are of course very limited and not representative. Nevertheless, we presented the raw data since they offer some exploratory insights and can be used to guide further research. Future investigations should focus on experimental approaches exploring the impact of informed consent and cover a wider array of mental health issues in children. In the future, a tailored approach for children and adolescents with autism spectrum disorder could offer a more authentic perspective. Additionally, integrating perspectives of parents and mental health professionals into the analyses may enhance understanding of the process.

## 6. Conclusions

This study is one of the few to examine minors’ views on confidentiality through interviews. We made an effort to include a diverse sample of both younger children and adolescents. Furthermore, we believe that this study highlights an important yet overlooked topic. Confidentiality is not self-explanatory, especially not for minors. Clear communication of confidentiality boundaries and respect for autonomy are crucial in mental health therapies for minors. Additionally, clarity in discussing sensitive topics like sexuality is crucial. This ensures minors feel acknowledged and receive appropriate developmental support, as well as mitigates negative treatment effects.

## Figures and Tables

**Table 1:** Participants.

Mean Age (in Years)	11.8
Country (*n*; %)	
Austria Germany	8; 72.7 2; 18.2
Non-EU countries	1; 9.1
Primary Diagnosis (*n*)	
Autism Spectrum Disorder—ASD	4
Attention-deficit/hyperactivity Disorder—ADHD	2
Affective Disorders	2
Psychosomatic Pain	1
Encopresis	1
Auto-immune Disorder	1

**Table 2:** Participants’ Perceptions of Confidentiality (*n* = 10).

	Number of Nominations		
Topic	Therapist Will Tell Parent	Therapist Will Not Tell Parent	Not Sure
Being hit by other kids	10	-	-
Not wanting to take medication	8	-	2
Taking drugs	7	2	1
Having been verbally abused by other children	7	1	2
Being pregnant	6	2	2
Not knowing whether to feel like a boy or a girl	6	-	4
Suicidal thoughts	6	3	1
Not wanting to live at home	6	2	2
Posting nasty pictures of others online	6	1	3
Receiving nasty text messages from other children	6	2	2
Having been teased by other children online	6	2	2
Hearing voices	5	2	3
Self-harming behaviour	5	3	2
Wanting to hurt someone else	5	4	1
Smoking	5	3	2
Having played naked with other kids	5	2	3
Lighting fire	4	2	4
Having been drunk	4	2	4
Being mad at parents	4	6	-
Being scared of someone	4	3	3
Having questions about contraception	3	3	4
Not wanting to talk to the psychologist	3	4	3
Having watched a forbidden film	3	4	3
Knowing that a friend wants to run away from home	3	4	3
Stolen money from parents	3	3	4
Having written bad things about others online	3	5	2
Having played forbidden computer games	3	3	4
Having cried in therapy	3	6	1
Having hurt others	3	2	5
Shop-lifting	2	3	5
Having lied to parents	2	6	2
Having made nude selfies	2	3	5
Knowing that a friend wants to do something forbidden	2	5	3
Talking bad about parents in therapy	2	6	2
Disclosing a secret of the parents	1	7	2
Having disclosed a secret	1	7	2
Having watched porn	1	5	4

Note: One child refused to participate in the card sorting task.

## Data Availability

Due to serious concerns about the anonymity of the data, as the main body of data contains interview transcripts that may be retraceable to individual patients, these data are not made available.
